# MiR-221 and miR-130a Regulate Lung Airway and Vascular Development

**DOI:** 10.1371/journal.pone.0055911

**Published:** 2013-02-08

**Authors:** Sana Mujahid, Heber C. Nielsen, MaryAnn V. Volpe

**Affiliations:** 1 Program in Cell, Molecular and Developmental Biology, Sackler School of Graduate Biomedical Sciences, Tufts University School of Medicine, Boston, Massachusetts, United States of America; 2 Department of Anatomy and Cell Biology, Tufts University School of Medicine, Boston, Massachusetts, United States of America; 3 Division of Newborn Medicine, Department of Pediatrics, Floating Hospital for Children at Tufts Medical Center, Boston, Massachusetts, United States of America; University of Pennsylvania School of Medicine, United States of America

## Abstract

Epithelial-mesenchymal interactions play a crucial role in branching morphogenesis, but very little is known about how endothelial cells contribute to this process. Here, we examined how anti-angiogenic miR-221 and pro-angiogenic miR-130a affect airway and vascular development in the fetal lungs. Lung-specific effects of miR-130a and miR-221 were studied in mouse E14 whole lungs cultured for 48 hours with anti-miRs or mimics to miR-130a and miR-221. Anti-miR 221 treated lungs had more distal branch generations with increased Hoxb5 and VEGFR2 around airways. Conversely, mimic 221 treated lungs had reduced airway branching, dilated airway tips and decreased Hoxb5 and VEGFR2 in mesenchyme. Anti-miR 130a treatment led to reduced airway branching with increased Hoxa5 and decreased VEGFR2 in the mesenchyme. Conversely, mimic 130a treated lungs had numerous finely arborized branches extending into central lung regions with diffusely localized Hoxa5 and increased VEGFR2 in the mesenchyme. Vascular morphology was analyzed by GSL-B4 (endothelial cell-specific lectin) immunofluorescence. Observed changes in airway morphology following miR-221 inhibition and miR-130a enhancement were mirrored by changes in vascular plexus formation around the terminal airways. Mouse fetal lung endothelial cells (MFLM-91U) were used to study microvascular cell behavior. Mimic 221 treatment resulted in reduced tube formation and cell migration, where as the reverse was observed with mimic 130a treatment. From these data, we conclude that miR-221 and miR-130a have opposing effects on airway and vascular morphogenesis of the developing lung.

## Introduction

Organogenesis in tubular organs is a complex process, requiring the coordinated branching and juxtaposing of the epithelial and blood vessel networks. Increasing evidence indicates that the developing vasculature tree plays an important role in guiding the branching pattern of many organs, including the lung [1]–[4]. The intimate association between the epithelial and endothelial cells during lung development is extremely important for establishment of the air-blood barrier to ultimately permit gas exchange at birth. However, very little is known about signaling mechanisms that govern this developmental process.

MicroRNAs (miRNAs) are important regulators of many biological processes in development and disease states. Mature miRNAs are produced by enzymatic processing of pre-miRNA sequences that involves the enzymes Drosha and Dicer. The mature miRNAs are small, non-coding RNAs that bind to the 3′ untranslated region or the coding region of target mRNAs to suppress translation. MiRNAs act as molecular switches, fine tuning the signaling for specific cellular events in development of several organs, including the lung. Several studies indicate that miRNAs are temporally and spatially regulated within developing organs, including the lung [5]–[8]. A targeted deletion of Dicer from developing lung epithelium caused severe alterations in lung development, including arrested branching and grossly dilated proximal and distal airways [9]. The mechanisms by which miRNAs regulate lung branching morphogenesis remain to be uncovered.

MicroRNAs are also crucial in regulating endothelial cell biology. Several studies describe promotion or suppression of angiogenesis by specific microRNAs, including miR-221 and miR-130a. MiR-221 is anti-angiogenic and decreases endothelial cell proliferation, migration and wound healing in cell culture models [10]–[12]. In contrast, miR-130 is pro-angiogenic, promoting endothelial cell proliferation and migration by inhibiting anti-angiogenic homeobox proteins [13]. Mir-221 and miR-130a are reported to target two Hox genes known to have important functions in embryonic lung branching morphogenesis and epithelial cell fate [14]–[21]. Hoxb5 has been identified as a target of miR-221 in thyroid carcinoma, and Hoxa5 as a target of miR-130a in human umbilical vein endothelial cells (HUVEC) cells [13], [22]. Further, both Hoxb5 and Hoxa5 have important regulatory roles in vascular development. Hoxb5 provides positive feedback to control the formation of angioblasts and maturation of endothelial cells [23]–[25]. In contrast, Hoxa5 upregulates expression of anti-angiogenic factors, thus promoting stabilization of blood vessels. Importantly, the opposing effects on angiogenesis of miR-221 and miR-130a and their respective targets Hoxb5 and Hoxa5, and the functions of Hoxb5 and Hoxa5 on airway morphogenesis suggests that these miRNAs and Hox proteins control both lung blood vessel and airway development.

We propose that miR-221 and miR-130a actively participate in regulation of embryonic lung vascular and airway branching. We used *ex vivo* and *in vitro* culture models to identify the phenotypic function of miR-221 and miR-130a in branching morphogenesis and neovascularization of the developing lung. We show that these two miRNAs have opposing effects on airway and vascular branching in the developing lung.

## Methods

### Animals

The animal study protocol was approved by the Tufts/Tufts Medical Center Institutional Animal Care and Use Committee (PHS Assurance A3775-01). Principles of laboratory animal care (National Institutes of Health publication 86–23, revised 1985) were followed. Timed-pregnant Swiss Webster mice were obtained from Charles River Laboratories (Wilmington, MA, USA), with the morning of the vaginal plug defined as embryonic day 0 (E0).

### Mouse Fetal Lung Endothelial Cell Line

MFLM-91U cells (ATCC, Rockport, MD) were maintained in Ultraculture medium (Lonza, Basel, Switzerland) with penicillin/streptomycin (50 units/µg), 1% Glutamine and 0.5% fetal calf serum (FCS) and cultured in 5% CO_2_, 37°C incubator [26].

### *In Situ* Hybridization

Fetal (E16–E18) lungs were fixed with 4% paraformaldehyde in phospho-buffered saline (PBS) (Lonza, Basel, Switzerland) at 4°C and transferred to 30% sucrose overnight. Lungs were embedded in OCT and stored at −80°C. The *in situ* hybridization protocol was adapted from a previously published protocol [27]. Briefly, sectioned lungs (N = 3 per gestational age) were fixed with 4% paraformaldehyde and digested with Proteinase K for 10 minutes. After incubation in prehybridization buffer, sections were hybridized with Locked Nucleic Acid probes (Exiqon, Vedbaek, Denmark) in 50% formamide hybridization mix at 53°C overnight. Slides were then washed with 5× SSC (Ambion, Grand Island, NY) and 0.2× SSC at 50°C. Slides were blocked with 10% FCS and incubated with anti-digoxigenin-alkaline phosphatase antibody (Sigma-Aldrich, St. Louis, MO) overnight, followed by color development (Vector, Burlingame, CA).

### Realtime PCR Analysis

Total RNA was extracted from E15– E18 whole fetal lungs, *ex vivo* lung cultures and endothelial cells using mirVana miRNA Isolation kit (Ambion, Grand Island, NY) according to manufacturer’s recommendations. Six animals from three different litters were used for each gestational age. For *ex vivo* cultures, two to three lungs were pooled per condition for each experiment. Real-time PCR for miRNAs was done using TaqMan microRNA Reverse Transcription Kit and TaqMan microRNA Assay (Applied Biosystems, Carlsbad, CA) according to manufacturer’s instructions. Three separate experiments were performed, each containing lungs from 3 litters.

### Whole Fetal Mouse Lung Organ Cultures

E14 whole fetal mouse lungs were removed intact and placed on Transwell 12 mm inserts (Corning, Lowell, MA) suspended at the air-liquid interface in Dulbecco’s Modified Eagle Medium (DMEM) with penicillin/streptomycin (50 units/µg) +10% charcoal-stripped FCS and cultured in 5% CO_2_, 37°C. At 24 hours of culture, lungs were randomly assigned for an additional 48 hours to the following conditions, which are summarized in Table 1: A) To inhibit specific miRNA function, antisense oligos (anti-miRs) to miR-221 or miR-130a (IDT, Coralville, IA), or anti-miR scrambled control oligo (Ambion, Grand Island, NY); B) To upregulate specific miRNA function, Pre-miR 221 (Mimic 221), Pre-miR 130a (Mimic 130a) or Pre-miR scrambled control (Ambion, Grand Island, NY) oligos. Anti-miRs and Pre-miRs were used at concentrations of 400 nM and 200 nM, respectively. Optimum experimental concentrations were determined by a dose-response assay. Lungs were cultured for 72 hours. All lungs were visually evaluated and photographed daily to quantify growth and airway branching (see below). At the end of the culture period, lungs were harvested and processed for morphometric, Real-time PCR, immunohistochemistry and immunofluorescence evaluations. Five separate experiments were performed, each containing lungs from 3 litters.

**Table 1:** Summary and explanation of all miR-221 and miR-130a manipulations used in experiments.

Molecule	Abbreviation	Function	Control
**Anti-miR-221**	Anti-221	DownregulatemiR-221	Anti-SCR
**Pre-miR-221**	Mimic 221	UpregulatemiR-221	SCR
**Anti-miR-130a**	Anti-221	DownregulatemiR-130a	Anti-SCR
**Pre-miR-130a**	Anti-130a	UpregulatemiR-130a	SCR

### Immunofluorescence and Immunohistochemistry

E14 explant lungs cultured for 72 hrs were used for whole-mount immunofluorescence (N = 3 per condition). The lungs were fixed in 4% PFA at 4°C, washed with PBS (Lonza, Basel, Switzerland) and dehydrated gradually up to 100% methanol. The whole mount immunohistochemistry protocol was adapted from Metzger et al. [28]. Briefly, samples were quenched in 5% H_2_O_2_/methanol and rehydrated gradually in PBST (0.5% Tween). They were blocked for 2 hours in 5% rabbit serum/PBST (Vector Labs, Burlingame, CA) and incubated for 2 days with E-cadherin (Zymed, Grand Island, NY) primary antibody at 4°C. Lungs were then washed in 5% rabbit serum/PBST and incubated with biotin-conjugated Rabbit anti-Rat IgG (Vector, Burlingame, CA) overnight at 4°C followed by sequential incubations in ABC Elite reagent (2 hours) (Vector, Burlingame, CA) and Flourescein Tyramide Reagent (Perkin Elmer, Waltham, MA). Samples were rinsed, mounted and stored in Vectashield (Vector, Burlingame, CA). Lungs were imaged using a Nikon A1R Confocal microscope at 20× magnification (Emission: 500–550 nm). Z-stacking through the entire thickness of the distal lung (based on the fluorescent signal) was performed using automated functions scanning at 5 µm intervals. Automated 6×6 panels where generated and stitched together using the NIS software (Nikon) to obtain an image that encompassed almost the entire lung area. Projections of these complete images were developed using the confocal software and used to visualize changes in airway branching.

Lung tissue sections were prepared from 4% PFA-fixed specimens embedded in OCT (N = 3 sections from ≥3 different experiments) as previously described and used for immunostaining. Briefly, coronal frozen tissue sections (8 µm) were immunostained using Avidin-Biotin (ABC) methodology (Vector, Burlingame, CA) with blue alkaline phosphatase detection. Primary antibodies used were: Hoxb5; Hoxa5 (Santa Cruz, CA); Sox2 (Chemicon, Billercia, MA); or Vascular Endothelial Growth Factor Receptor type 2 (VEGFR2) (Cell Signaling, Danvers, MA); To confirm the specificity of staining for each protein, immunostaining controls included absence of primary antibody and reaction of separate tissue sections with different primary antibodies in the same immunostaining reaction. To allow direct comparisons of the evaluated proteins between the Control and Experimental Groups, immunostaining reactions were performed simultaneously and incubated for identical times for the chromagen detection step.

Endothelial cells were identified in frozen sections by staining with GSL-B4 lectin (3 sections each from ≥3 different experiments). Sections were hydrated in PBST and incubated with Fluorescein GSL-B4 lectin (Vector, Burlingame, CA) for 25 minutes, and mounted using Dapi Vectashield (Vector, Burlingame, CA).

### Lung Morphometry (Point Counts)

Lung morphometry was measured to quantify phenotypic changes in lung morphology as we have previously described [29]. Using a computer generated 25 µM grid overlayed on a 20× magnification of selected tissue sections (separated by at least 18µM), airway space, mesenchyme, and epithelial regions were identified at each intersecting point on the grid (point counts). The percentage mean of summed point counts of airway space, mesenchyme and epithelial cells from each tissue section were compared between Control and Experimental groups (N = 3 lungs per condition).

### Quantification of Branching Changes in Whole Lung Cultures

To confirm and enhance the assessment of changes in the lung branching phenotype, we measured branch width in the E-Cadherin stained lungs using NIS software (Nikon, Melville, NY) (method adapted from Lazarus et al [4]). The branch width was measured at terminal branch tips. At least 10 different branch width measurements were obtained from three randomly chosen regions for each lung (N = 3 lungs per condition).

### Matrigel Angiogenesis Assay

A dose response was done in MFLM-91U cells to confirm that 1 nM of Mimic upregulated miR-221 levels by 100 fold and miR-130a levels by 20 fold. MFLM-91U cells were transfected with 1 nM of scrambled, Mimic 221 or Mimic 130a and cultured for 24 hours. After trypsinization, cells were counted and plated on a thin layer of growth factor reduced matrigel (BD Biosciences, San Jose, CA) in 24-well plates (10^6^ cells/well). Pictures under a 10× magnification were taken after 5 hours of culture. The number of tubes, defined as cell projections that connect two cell bodies was counted per 10× magnification field using NIS software (Nikon) [13]. Pictures of at least 3 different fields were taken per condition for each experiment (N ≥3).

### *In vitro* Scratch Assay

MFLM-91U cells were grown to confluence on 12-well plates and transfected with 1 nM Mimic 221 and 130a for 36 hours, after which they were scraped with a sterile P200 tip to generate a cell-free zone. Wells were washed with PBS, retransfected and incubated for another 18 hours. Photographs of at least 3 different fields per condition were taken for each experiment (N = 4) under 10× magnification. The width of the cell-free scratched area was measured at the time of injury and after 18 hours, and the difference calculated [30].

### Statisical Analysis

Statistical analysis was done using non parametric ANOVA or two-tailed t-tests (GraphPad Software, San Diego, CA) as appropriate with a level of significance of P<0.05. The data are expressed as Mean±SEM.

## Results

### miR-221 and miR-130a are Temporally and Spatially Regulated Across Gestation

**Figure 1:**
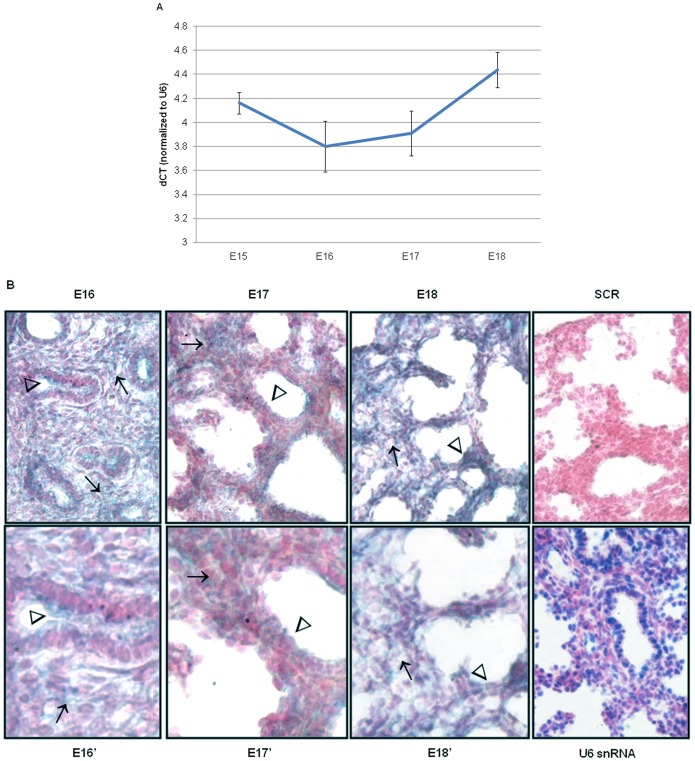
miR-221 temporal, spatial and cellular localization changes with advancing gestation. (A) Quantification of total miR-221 levels in mouse fetal of gestational days E15– E18 was done by qRT-PCR. ΔCT values were calculated using U6 snRNA as an internal control. Mean ± SEM of N = 6 per gestation. (B) *In situ* hybridization was done using DIG-labeled miR-221 probes on frozen sections obtained from E16– E18 mouse fetal lungs. U6 probes were used as a positive control and scrambled probes were used as a negative control. Sections were counterstained using Fast Red. E16–E18, SCR, and U6 snRNA images were taken at 40× magnification. E16′–E18′ are 80× magnification (N = 3; arrows point to mesenchyme; arrowheads point to epithelium).

To investigate the temporal and spatial expression of miR-221 and miR-130a in the developing lung, qRT-PCR and *in situ* hybridzation were done on mouse fetal lungs. Total miR-221 expression initially decreased from E15 to E16, followed by an increase as gestation progressed (Figure 1A). MiR-221 cellular expression was observed in epithelial and mesenchymal compartments at E16 and E17 on *in situ* evaluation, but the spatial localization changed with advancing gestation (Figure 1B). At E16, miR-221 was intensely expressed in bronchiolar epithelium (arrowhead) and mesenchyme (arrows) whereas at E17 mesenchymal expression remained strong (arrow) and epithelial expression decreased (arrowhead). Mesenchymal expression became more diffuse at E18 (arrow) compared to E16 and E17 and epithelial expression was only minimally present (arrowhead).

**Figure 2:**
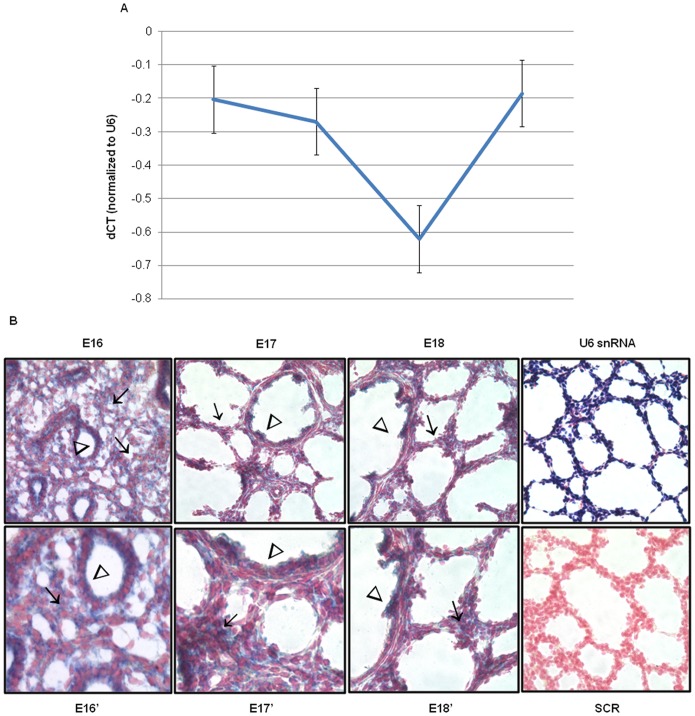
miR-130a temporal, spatial and cellular localization changes with advancing gestation. (A) Quantification of total miR-130a levels in gestational days E15– E18 mouse fetal lungs was done by qRT-PCR. ΔCT values were calculated using U6 snRNA as an internal control. Mean ± SEM of N = 6 per gestation. (B) *In situ* hybridization was done using digoxigenin-labeled miR-130a probes on frozen sections obtained from E16– E18 mouse fetal lungs. U6 probes were used as a positive control and scrambled probes were used as a negative control. Sections were counterstained using Fast Red. E16–E18, SCR, and U6 snRNA images were taken at 40× magnification. E16′–E18′ are 80× magnification (N = 3; arrows point to mesenchyme; arrowheads point to epithelium).

Total miR-130a expression levels decreased at E16 and E17 before increasing at E18 to levels seen at E15 (Figure 2A). MiR-130a localization also changed with advancing gestation (Figure 2B). Different from miR-221, miR-130a expression was more intense in the epithelium (arrowhead) compared to the mesenchyme at E16 (arrows). Certain clusters of mesenchymal cells (arrow) and of the bronchiolar epithelium (arrowheads) were strongly positive at E17. By E18, miR-130a was restricted to the terminal bronchioles (arrowhead) and mesenchymal cell clusters around developing saccules (arrow). Taken together, analysis of miR-221 and miR-130a distribution in the developing lung suggests functional roles in the progression of lung development.

### miR-221 and miR-130a Regulate Branching Morphogenesis

**Figure 3:**
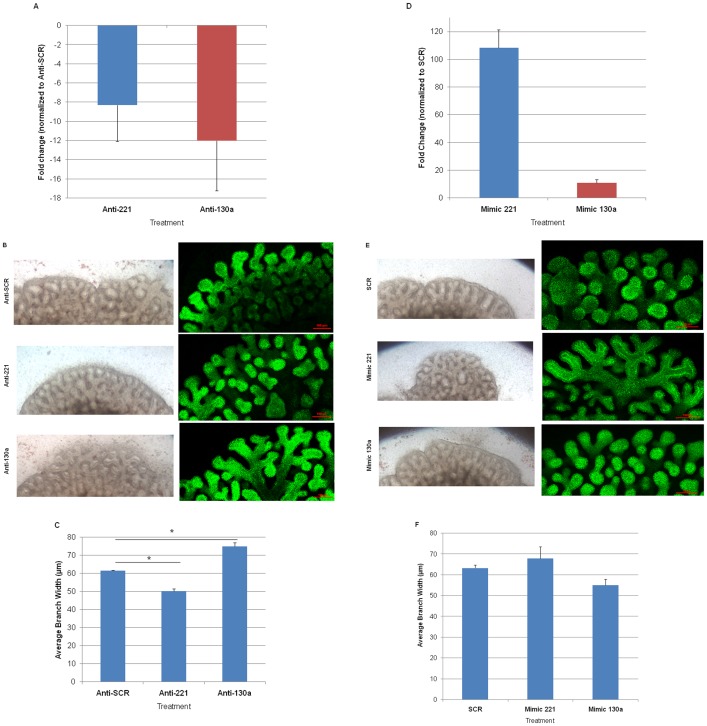
Treatment of lung organ cultures with anti-miRs and mimics alters airway branching. E14 mouse fetal lungs were harvested and cultured on porous membranes for 72 hours. (A) Lungs were transfected the day after harvest with anti-SCR, anti-221 or anti-130a. Downregulation of miR-221 (blue bar) and miR-130a (red bar) was confirmed by qRT-PCR. Mean ± SEM of N = 3 experiments. (B) Brightfield images were taken after 24 hours of treatment. After 48 hours of treatment, lungs were fixed and stained for E-cadherin. Z-stack confocal images of whole mount E-cadherin stained lungs were obtained and the final image of the entire lung obtained by software combination of the z-stacks. Altered airway branching is seen after 24 and 48 hours after transfection. Anti-221 lungs have increased proximal and distal airway branching, and narrower terminal branch tips. Anti-130a lungs have wider and fewer airway branches. (C) Width of distal airway tips was measured for each condition and compared to Anti-SCR. Mean ± SEM of N ≥3, *p<0.01. (D) Lungs were transfected the day after harvest with SCR, Mimic 221 or Mimic 130a. Upregulation of miR-221 (blue bar) and miR-130a (red bar) was confirmed by qRT-PCR. Mean ± SEM of N = 3 experiments. (E) Brightfield images were taken after 24 hours of treatment. After 48 hours of treatment, lungs were fixed and stained for E-cadherin. Z-stack confocal images of whole mount E-cadherin stained lungs were obtained and the final image of the entire lung obtained by software combination of the z-stacks. Mimic 221 lungs had decreased airway branching whereas mimic 130a had increased central and peripheral branching. (F) Width of distal airway tips were measured for each condition and compared to SCR. Mean ± SEM of N ≥3.

To begin to understand the functional roles of miR-221 and miR-130a in the developing lung, E14 *ex vivo* lung cultures were treated with anti-miRs to downregulate or mimics to upregulate these miRNAs. Changes in total miR-221 and miR-130a were verified by qRT-PCR to ensure transfection efficiency (Figure 3A, 3D). Lungs treated with anti-221 and anti-130a showed altered lung branching after 24 hours in culture (Figure 3B, left panel), which became more evident after 48 hours (Fig. 3B, right panel; z-stack images of entire cultured lungs obtained by confocal microscopy). Compared to the anti-SCR, E-Cadherin-staining of anti-221 treated lungs identified more distal branch generations whereas anti-130a treated lungs had decreased branching. Z-stack images showed that the increase and decrease in branching was reflected by 3-dimensional changes in branching morphology.

The opposite phenotypes were observed when lungs were transfected with mimics to miR-221 and miR-130a. Compared to SCR controls, mimic 221 treated lungs had dilated airway tips and fewer airway branches (Figure 3E; right panel shows z-stack images of entire cultured lungs obtained by confocal microscopy). These branches also appeared to have increased interbud airway length. Mimic 130a treated lungs appeared to have increased airway branching with decreased branch width (Figure 3E). Again, the z-stack images illustrate that the increase and decrease in branching was reflected by 3-dimensional changes in branching morphology. Whole lung brightfield and E-cadherin images for anti-miR and mimic treated lungs are included in Figure S1.

To further characterize and quantify changes in airway branching that were visually observed, the width of terminal branch tips was measured. Decreased branching and wider airways are consistent with proximalization of branch phenotype, and vice versa [31]. The branch width of anti-221 treated lungs decreased compared to anti-SCR lungs In contrast, anti-130a lungs had increased branch width (Figure 3C; P<0.01, Mean ± SEM; N≥3). The corresponding opposite branching phenotype was observed for each miRNA when lungs were treated with mimics to upregulate miRNA expression levels. Mimic 221-treated lungs showed a trend towards increased branch width while mimic 130a-treated lungs exhibited reduced branch width (Figure 3F; N≥3). Overall, the results of anti-miR and mimic treatments suggest that both miRNAs are involved in lung airway branching.

### Effect of miR-130a and miR-221 on Epithelial Cell Fate And Morphology

**Figure 4:**
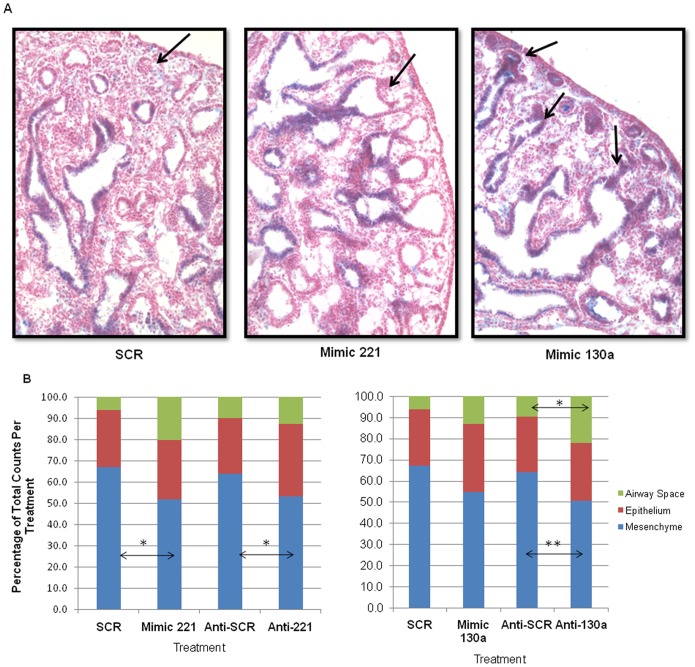
Epithelial and morphological changes observed in lungs with anti-miR and mimic treatments. (A) Mimic 130a alters epithelial expression of Sox2, a transcription factor expressed in proximal but not distal airways. Immunohistochemistry was done on sections of cultured lung using Sox2 antibody. Mimic 130a lungs had nuclear Sox2 expression in distal airway epithelium (arrows), which was minimal or absent in SCR and Mimic 221 lungs. Images were taken at 20× magnification. The figure shows representative images from N = 3 lungs per condition. (B) Morphometric analysis showed altered lung morphology in mimic and anti-miR-treated lungs. Point count analysis was done by placing a 50μm grid on histological sections. Mimic 221 had a 15.2% decrease and anti-221 a 10.5% decrease in mesenchymal area compared to controls. Mimic 130a treatment showed a trend towards decreased mesenchymal area and an increase in epithelial and airway space area. Anti-130a treatments caused a 13.4% decrease in mesenchymal area and a 12.4% increase in airway space area. Mean ± SEM of N ≥3, * p<0.05, **p<0.01. N≥40 tissue sections each separated by at least 18µm from ≥3 lungs per condition.

Some of the opposing airway branching phenotypes created by perturbations of miR-221 and miR-130a could be due to regulation of an epithelial mechanism controlling the progression of branching morphogenesis. To explore this possibility, we evaluated whether miR-130a and miR-221 altered epithelial progenitor cell fate. Lung sections were stained for Sox2, which is present in endodermal epithelial progenitors of proximal airways and absent in distal airways [32], [33]. Compared to SCR, Mimic 130a treated lungs had numerous distal airways in the periphery of the lung that stained positive for Sox2 The opposite phenotype was observed in anti-130a treated lungs (data not shown). However, no change in Sox2 expression was observed in Mimic 221 treated lungs (Figure 4A; arrows point to distal epithelium). These studies suggest that miR-130a may be involved in expanding proximal progenitors in the developing lung.

Morphometric point counts were used to further characterize changes in gross branching phenotype and lung histology seen in tissue sections. The analysis of lung sections from anti-221 and mimic 221 treatments showed a significant decrease in mesenchymal volume and a trend towards increased airway space volume in both instances (Figure 4B; P<0.05). Anti-221 treated lungs had more airways where as mimic 221 lungs had fewer, more dilated terminal airways. The opposing phenotypes in airway morphology explain the similar directionality of changes in mesenchymal and airway space volume with the two treatments. A similar trend was observed with anti-130a and mimic 130a transfected lungs. Both treatments resulted in decreased mesenchymal and increased airway space volume, with changes observed with mimic 130a being significant (Figure 4B; P<0.01 and P<0.05, for mesenchyme and air space, respectively). These results are consistent with fewer airways in anti-130a treated lungs, whereas more airway branches were observed with mimic 130a treatment.

### miR-221 and miR-130a Target Expression of Hox Proteins

**Figure 5:**
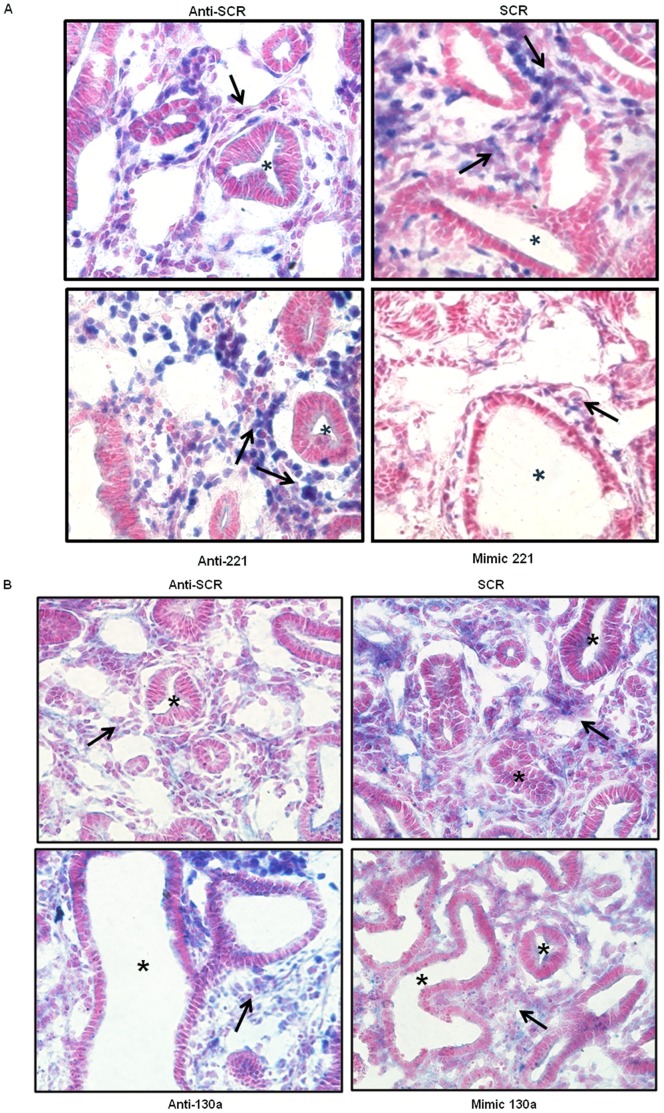
miR-221 and miR-130a alter localization of Hox protein expression. (A) Manipulation of miR-221 levels alters Hoxb5 localization. Immunohistochemical analysis of Hoxb5 localization was done on anti-miR and mimic 221 treated lung sections. Intensity of Hoxb5 nuclear expression in anti-221 treated lungs was increased in mesenchyme (arrows) around airways (asterisk) compared to anti-SCR control. Decreased Hoxb5 expression (arrows) was seen in mimic 221 treated lungs compared to SCR. (B) Manipulation of miR-130a levels alters Hoxa5 localization. Immunohistochemistry analysis of Hoxa5 localization was done on anti-miR and mimic 130a treated lung sections. Increased Hoxa5 expression (arrows) was present in the mesenchyme of anti-130a treated lungs, whereas Hoxa5 expression was decreased in mimic 130a treated lungs compared to controls. Images were taken at 40× magnification (N = 3 lungs per condition; arrows point to mesenchyme; asterisks mark airways).

Hoxb5 is a target of miR-221 in human thyroid carcinoma cells. Therefore, we investigated how miR-221 manipulation impacted Hoxb5 expression patterns within the developing lung [22]. We found that nuclear Hoxb5 mesenchymal expression in anti-221 treated lungs (Figure 5A, lower left panel, arrows) was more diffusely located compared to the anti-SCR control lungs (Figure 5A, upper left panel). Conversely, less intense nuclear Hoxb5 mesenchymal expression was seen in mimic 221 treated lungs (Figure 5A, lower right panel, arrow) compared to the SCR control (Figure 5A, upper right panel). The increased dilation of the airways caused by mimic 221 (Figure 5A, asterisk in upper and lower right panels) is a phenocopy of Hoxb5 downregulation [34]. Exogenous miR-130a has been shown to reduce Hoxa5 protein expression in HUVEC cells [13]. Compared to anti-SCR treated lungs (Figure 5B, upper left panel arrow), treatment with anti-130a resulted in a more diffusely localized mesenchymal Hoxa5 cellular expression pattern (Figure 5B, lower left panel, arrow). Less intense Hoxa5 mesenchymal cellular localization was observed with mimic 130a treatment (Figure 5B, lower right panel, arrow) compared to the SCR control (Figure 5B, upper right panel, arrow). These results suggest a regulatory role for miR-221 and miR-130a on Hoxb5 and Hoxa5, respectively, in developing lung.

### miR-221 and miR-130a Regulate Neovascularization During Lung Branching Morphogenesis

**Figure 6:**
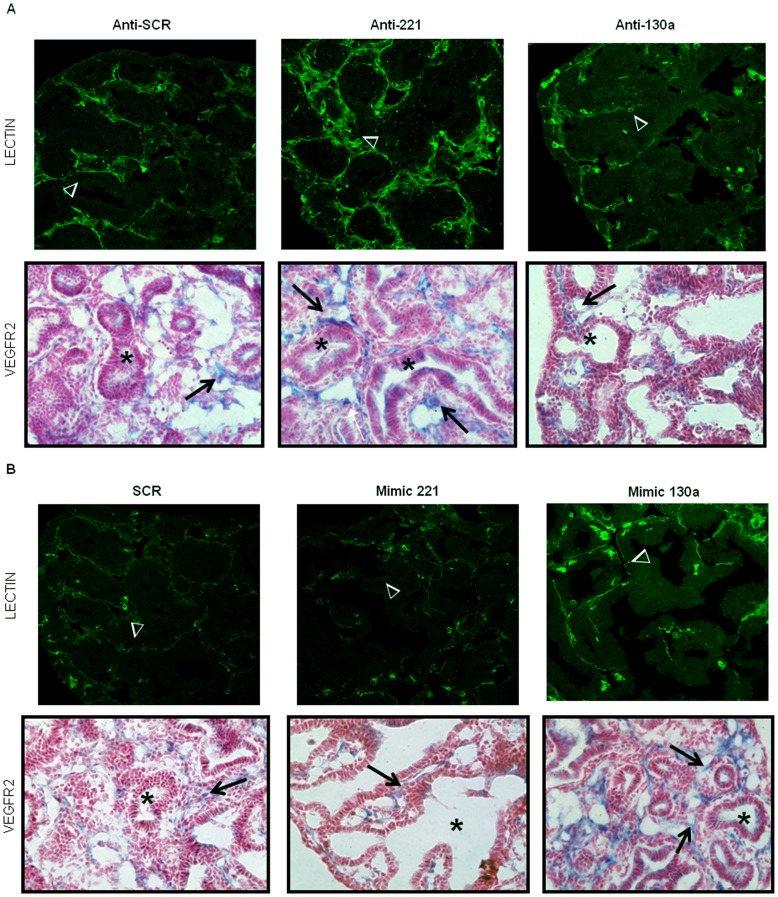
Altered vascular morphology in anti-miR treated E14 lung buds. Lectin immunofluorescence and VEGFR2 immunohistochemistry were done on lung sections to assess vascular changes. (A) Compared to anti-SCR controls lectin staining of anti-221 treated lungs (arrowheads) showed a much more robust vascular network around developing airways. These airways were also surrounded by cells subjacent to the epithelium which were strongly positive for VEGFR2 (arrows). In contrast, lectin (arrowheads) and VEGFR2 staining (arrows) of anti-130a treated lungs showed small vascular tufts around distal airway branches (asterisk). (B) Mimic 221 treatment led to a disorganized vascular network as seen by lectin (arrowheads) and VEGFR2 staining (arrows). Conversely, lectin staining (arrowheads) of mimic 130a treated lungs showed a more vascularized network within the lung. These lungs also had more airways (asterisk) encircled by VEGFR2 receptor positive cells subjacent to the epithelium (arrows). Images were taken at 40× magnification (N = 3 lungs per condition; arrowheads point to vessels; arrows point to mesenchyme; asterisks mark airways).

Many studies strongly indicate that epithelial branching in the developing lung is partially controlled by the development of the pulmonary vascular bed [1]–[4]. Several groups have shown that miR-221 and miR-130a regulate endothelial cell function, suggesting that regulation of these miRNAs in the embryonic lung vasculature may also affect airway branching morphogenesis [10]. MiR-221 has anti-angiogenic properties through targeting several important proteins involved in angiogenesis. MiR-130a, on the other hand, is pro-angiogenic. Given that upregulating miR-221 or downregulating miR-130a (and vice versa) in *ex vivo* lung cultures produce a similar branching phenotype, we wanted to elucidate whether miR-221 and miR-130a had opposing effects on the underlying vascular bed in these lungs as well. Transfected lung organ culture tissue sections were stained with fluorescein-labeled endothelial cell GSL-B4 lectin and VEGFR2 antibody to visualize the lung vasculature. Lectin staining of anti-221 treated lungs demonstrated a more robust vascular network around developing airways in both central and peripheral lung compared to the SCR controls (Figure 6A, arrowheads). The airways (asterisks) were also surrounded by cells beneath the airway epithelium which were strongly positive for VEGFR2 (arrows). In contrast, lectin staining of anti-130a treated lungs identified the presence of small vascular tufts around distal airway branches; most of the vascular network was poorly developed and not well defined (Figure 6A, arrowheads). Few airways (asterisk) were surrounded by VEGFR2-positive cells under the airway epithelium. Most of the VEGFR2 was localized around airways at the edge of the lung (arrow).

Changes in the vascular bed of mimic 221 and mimic 130a treated lungs showed an opposite phenotype to their respective anti-miR treatments. Mimic 221 treatment led to the formation of a disorganized vascular network (arrow) surrounding large dilated airways (Figure 6B). There was a paucity of VEGFR2 positive cells (arrows) beneath the epithelium of these abnormal airways (asterisk). Conversely, mimic 130a treatment resulted in lungs with a more vascularized network. These mimic 130a treated lungs also had more airways encircled by VEGFR2 positive cells subjacent to the airway epithelium (Figure 6B, arrows). These results suggest that miR-221 and miR-130a target the developing lung vasculature and are consistent with reports that miR-221 is anti-angiogenic and mir-130a is pro-angiogenic. These observations provide insight into the mechanisms underlying similarities and differences in airway branching observed with miR-221 and miR-130a manipulation.

### miR-221 and miR-130a Target Angiogenesis in vitro

**Figure 7:**
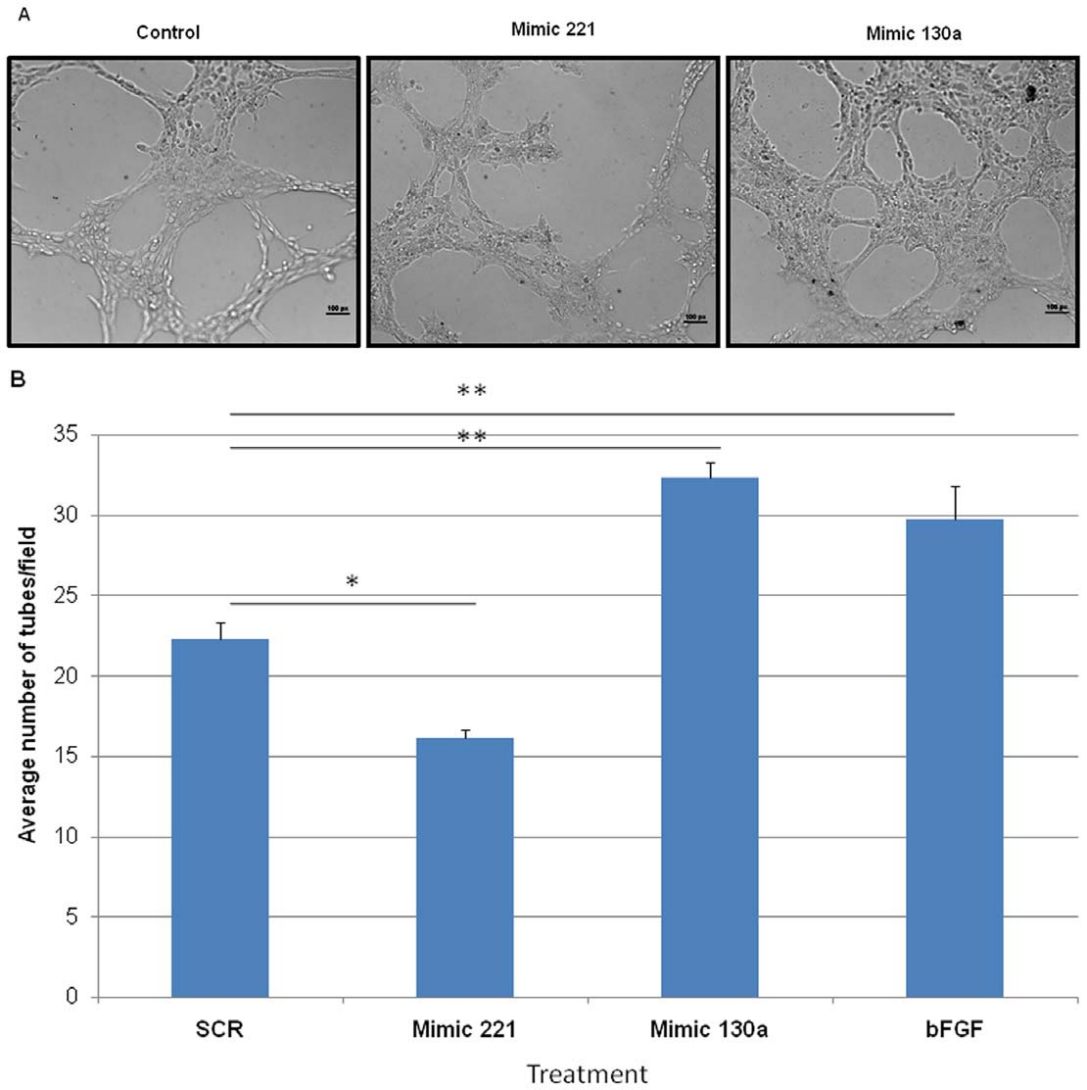
Effects of mimic 221 and mimic 130a on in vitro angiogenesis. Mouse fetal lung endothelial cells were transfected with SCR, mimic 221 or mimic 130a 24 hours prior to being plated on matrigel for an additional 16 hours. (A) Compared to control, mimic 221 treatment inhibited tube development and limited endothelial cell projections. In contrast, mimic 130a treated cells had more developed cellular projections forming an extensive tubular network and vascular plexus. (B) Tubes were quantified per low power field. bFGF was used as a positive control. N ≥3, * p<0.05, **p<0.01. Error bars represent SEM.

**Figure 8:**
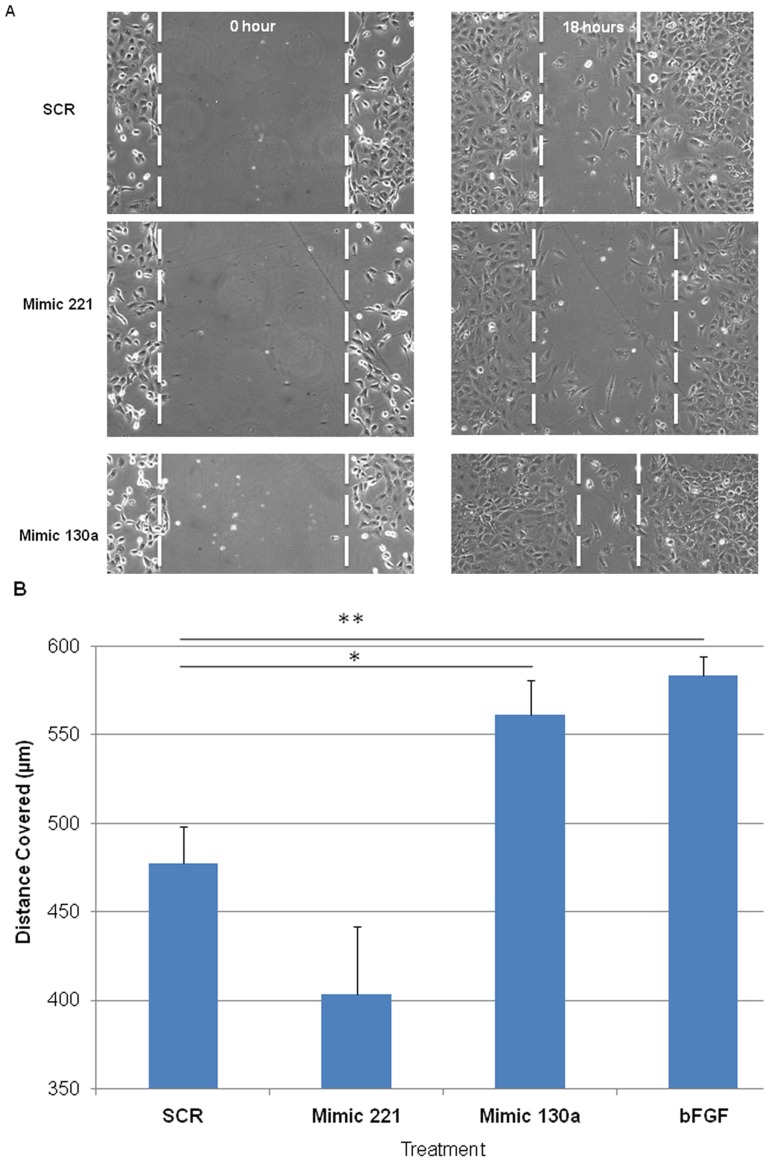
Mimic 221 and mimic 130a alter endothelial cell migration in vitro. Transfected endothelial cells were grown to confluence and a scratch was made using a P200 tip to create a cell-free zone. Pictures were taken at the time the scratch was made and after 18 hours of incubation. (A) After 18 hours, mimic 221-transfected endothelial cells migrated at a slower rate, whereas mimic 130a-transfected endothelial cells migrated at a faster rate. (B) The width of the original scratch was measured at time 0 and subtracted from the width at 18 hours to calculate the distance covered. Compared to SCR, the rate of wound closure decreased with mimic 221 treatment, whereas it increased with mimic 130a transfection. bFGF was used as a positive control. N≥4 * p<0.05, **p<0.01. Error bars represent Mean ± SEM.

To address whether miR-221 and miR-130a altered neovascularization by directly targeting fetal lung endothelial cells, we utilized an *in vitro* angiogenesis assay. Mouse lung fetal endothelial cells (MFLM-91U) transfected with mimic 221, mimic 130a or SCR oligos were plated on a thin layer of matrigel and incubated for 5 hours. Upregulation of miR-221 and miR-130a was confirmed by qRT-PCR (60 to 100 fold up regulation, data not shown). Cells treated with mimic 221 had limited endothelial cell projections and reduced tube formation compared to SCR treated cells (Figure 7A, B; P<0.05 in 7B). In contrast, mimic 130a treated cells had more developed cellular projections forming an extensive tubular network with an increase in plexus formation (Figure 7A, B; P<0.01 in 7B). It has previously been shown that these two miRNAs can affect endothelial cell migration. We used an in vitro scratch assay to observe the effect of miR-221 and mir-130a on endothelial cell migration. Efficiency of wound closure was increased in mimic 130a transfected cells, whereas mimic 221 slightly inhibited this process (Figure 8A, B; P<0.05 for mimic 130a). These studies show that miR-221 and miR-130a can alter vascularization by directly affecting endothelial cell behavior resulting in changes in tube formation and cell migration.

## Discussion

Airway branching morphogenesis is a complex process requiring coordinated interdependent signaling between different cellular compartments, namely the mesenchyme, endothelium and epithelium of the developing lung. This process enables the airways and vasculature to develop in a parallel and coordinated fashion. From recent advances in the understanding of miRNA biology, it is clear that miRNAs play important roles in lung morphogenesis, but our knowledge of how they regulate airway and vascular branching morphogenesis is extremely limited. Several studies have highlighted the role of miR-221 and miR-130a in regulating endothelial cell biology for angiogenesis, showing that miR-221 has angiostatic and miR-130a has pro-angiogenic properties [10]. The majority of these studies used *in vitro* HUVEC cell cultures. Therefore, very little is known about how these miRNAs function in organ development and nothing is known concerning their roles in coordinating lung airway and vascular formation.

Our present study used *ex vivo* and *in vitro* models to explore how miR-221 and miR-130a regulate airway and vascular branching in the lung. In this study we show for the first time that miR-221 and miR-130a regulate both airway branching and lung microvascular development. Our data show that the expression of both miRNAs is tightly regulated across gestation in the lung. Given the importance of balanced expression and function between molecules that promote and inhibit angiogenesis in development, it is not surprising that the temporal expression of miR-221 and miR-130a is finely tuned in the developing lung. We propose that these two miRNAs regulate airway development by, in part, targeting the underlying vasculature. These two miRNAs induced unique and opposing phenotypes when manipulated in *ex vivo* lung cultures, as summarized in the table below. These results are consistent with the current knowledge that miR-221 is anti-angiogenic and miR-130a is pro-angiogenic. Both downregulation of miR-221 and upregulation of miR-130a resulted in increased vascular density, with a concomitant increase in distal airway branching (Table 2). Furthermore, the opposite effects were observed with miR-221 upregulation or miR-130a downregulation. These treatments resulted in fewer blood vessels with fewer and wider terminal branches. These findings suggested that these miRNAs targeted lung endothelial cells and perturbed epithelial – endothelial interactions. Indeed, upon further investigation we found that miR-221 and miR-130a have opposing roles in vessel formation in developing lung vasculature, consistent with the current literature. Using mouse fetal lung endothelial cells we showed a direct effect of these miRNAs on key endothelial cell behaviors. MiR-221 inhibited endothelial cell tube formation and migration, whereas miR-130a enhanced tube and vascular plexus formation and cell migration. These observations paralleled the morphologic changes in the microvascular bed of *ex vivo* lung cultures, wherein treatment with mimic 221 resulted in disorganized vessel formation and mimic 130a treated lungs had a robust vascular network. These *ex vivo* and *in* *vitro* studies show that miR-221 and miR-130a mediate their effects on lung development partly through targeting the developing vasculature.

**Table 2:** Summary of phenotypes observed when *ex vivo* lung cultures were treated with anti-miRs or mimics to miR-221 and miR-130a.

Phenotype	Anti-221	Mimic 130a	Anti-130a	Mimic 221
**Number of Branches**	**↑**	**↓**	**↓**	**↓**
**Branch Width**	**↓**	**↓**	**↑**	**↑**
**Lectin**	**↑**	**↑**	**↓**	**↓**
**VEGFR**	**↑**	**↑**	**↓**	**↓**

Though the interrelationship between the developing vascular tree and airway epithelium is recognized, the mechanisms and overall contribution of the endothelium to the developing lung remains to be understood. Alterations in pulmonary vascular assembly have been shown to alter airway branching morphogenesis [1], [2]. Addition of recombinant vascular endothelial growth factor (VEGF), a promoter of vasculogenesis, to embryonic lung cultures resulted in a highly branched organ, while knocking down the VEGF receptor Flk-1 caused a significant decrease in airway branching morphogenesis [2]. The highly branched phenotype is similar to what was observed with miR-221 downregulation and miR-130a upregulation in the *ex vivo* lung cultures. Besides regulating the degree of airway branching, the vasculature also determines branching stereotypy and is important for the 3D patterning of the lung [4]. We found that increased miR-221 or decreased miR-130a levels in lung cultures produced a disorganized vascular network that was associated with reduced airway branching. These branches were also dilated, a phenotype that is observed with overexpressing the VEGFR1 decoy receptor in the lung as well as knock down of the Flk-1 receptor [2], [4]. These findings provide new insight into the mechanistic relationship between vascular and airway branching. This is the first study to show that miRNAs are involved in the crucial process of coordinated airway and blood vessel development.

Our findings with regards to the angiostatic properties of miR-221 and pro-angiogenic properties of miR-130a are consistent with studies of these miRNAs in human venous or lymphatic endothelial cells [11]–[13], [35]. However, miR-221 overexpression in lymph endothelial cells did not result in the migration defects that we observed using lung microvascular endothelial cells [35]. A recent study also found that miR-221 promotes endothelial tip cell migration in the development of zebrafish intersegmental vessels [36]. These findings suggest that miRNAs work in a cell- and organ- specific manner. The cellular context, along with the developmental stage, dictates miRNA targets and function. MiR-221 and miR-130a are present in multiple cellular compartments during lung development, as shown by the *in situ* hybridization results. The function of miR-221 and miR-130a in these other cell types is unknown and requires further investigation.

Manipulation of miR-221 and miR-130a levels caused spatial and cellular changes in Hoxb5 and Hoxa5 localization, respectively. While significant changes in whole tissue Hoxb5 and Hoxa5 protein levels were not observed (data not shown) this does not rule out regulatory relationships. It is important to note that subtle changes in Hox cellular expression patterns can dramatically impact tissue development [37], [38]. This suggests that changes in cellular localization of these Hox proteins with miRNA manipulation are biologically important in our model. This concept is further supported by our previously published work on the expression and function of these Hox proteins in the developing lung and the phenotype observed when miR-221 and miR-130a are manipulated [16], [34]. However, we have not demonstrated *cis*-regulation of Hoxb5 and Hoxa5 by these miRNAs. In an effort to look for specific targets of the two miRNAs in fetal lung endothelial cells, we used a vasculogenesis-focused protein array panel (R&D systems, Minneapolis, MN). We were unable to identify direct targets for either miR-221 or miR-130a in the lung endothelial cells. Reported targets of miR-221 and miR-130a in other cell types mostly include downstream signaling molecules and transcription factors which were not present in this array [39], [40]. In particular, despite the fact that the number of VEGFR2 immunopositive cells changed as a result of miRNA manipulations in our *ex vivo* cultures, we found no direct effect of either miRNA on VEGFR2 expression in lung endothelial cells (data not shown). This can be explained by the fact that miRNA manipulations caused changes in total VEGFR2 expressing endothelial cells in the *ex vivo* cultures and did not target VEGFR2 directly (data not shown). The direct targets of both miRNAs vary depending on the cell type. Identifying the targets of these miRNAs in fetal lung endothelial cells will help elucidate important components of the mechanisms controlling lung vascular and airway branching morphogenesis.

In summary, we have shown that miR-221 and miR-130a coordinately regulate airway and vascular branching. This function may involve control of epithelial – endothelial interactions in the developing lung. These studies create an important foundation for the possibility of miRNAs regulating epithelial – endothelial interactions during lung airway and vascular development.

## Supporting Information

Figure S1 **Whole lung images of brightfield and E-cadherin stained anti-miR and mimic treated lungs.** Representative brightfield images are shown on the left and Z-stack confocal images of whole mount E-cadherin stained lungs are shown on the right. Brightfield images were taken after 24 hours of treatment. After 48 hours of treatment, lungs were fixed and stained for E-cadherin. Z-stack confocal images of whole mount E-cadherin stained lungs were obtained and the final image of the entire lung obtained by software combination of the z-stacks. (A) Anti-SCR (B) Anti-221 (C) Anti-130a (D) SCR (E) Mimic 221 (F) Mimic 130a.
(TIF)
