# Supplementary figures and images for: MiR-221 and miR-130a Regulate Lung Airway and Vascular Development
Source: PLoS One. 2013 Feb 8;8(2):e55911. doi: 10.1371/journal.pone.0055911 (PMC3568032; doi:10.1371/journal.pone.0055911)

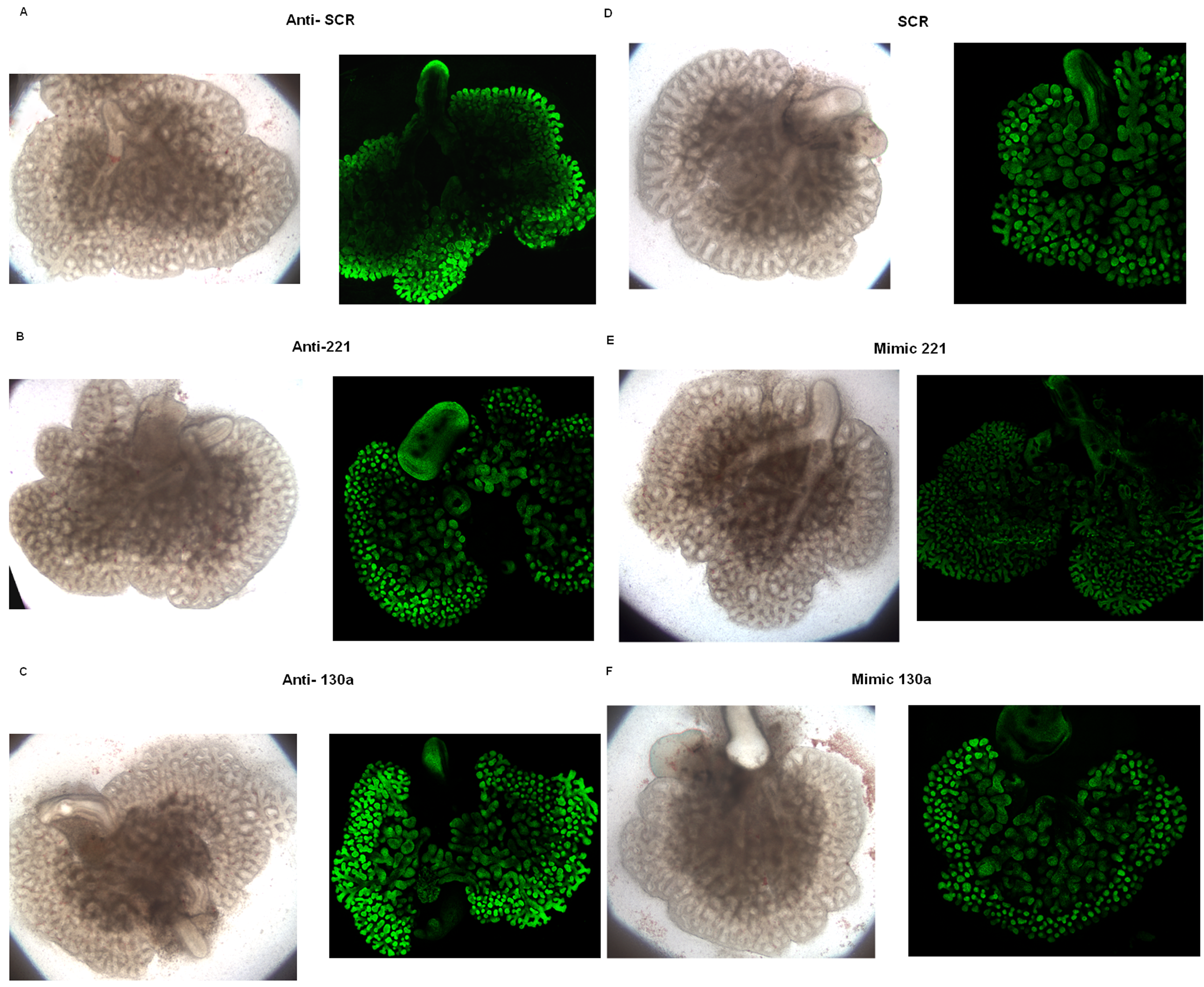

Supplement: Figure S1 — Whole lung images of brightfield and E-cadherin stained anti-miR and mimic treated lungs. Representative brightfield images are shown on the left and Z-stack confocal images of whole mount E-cadherin stained lungs are shown on the right. Brightfield images were taken after 24 hours of treatment. After 48 hours of treatment, lungs were fixed and stained for E-cadherin. Z-stack confocal images of whole mount E-cadherin stained lungs were obtained and the final image of the entire lung obtained by software combination of the z-stacks. (A) Anti-SCR (B) Anti-221 (C) Anti-130a (D) SCR (E) Mimic 221 (F) Mimic 130a. (TIF) [file pone.0055911.s001.tif]
